# Awareness of Racial and Socioeconomic Health Disparities in the United States: The National Opinion Survey on Health and Health Disparities, 2008-2009

**Published:** 2011-06-15

**Authors:** Bridget C. Booske, Stephanie A. Robert, Angela M. K. Rohan

**Affiliations:** University of Wisconsin-Madison, Department of Population Health Sciences; University of Wisconsin-Madison, Madison, Wisconsin; Wisconsin Department of Health Services, Madison, Wisconsin

## Abstract

**Introduction:**

Recent initiatives aim to improve public awareness of health disparities. However, little research has actually documented the US public's awareness of racial/ethnic and socioeconomic health disparities. We sought to determine 1) whether the US public is aware of racial, educational, and income disparities in health, 2) whether awareness differs across these disparity domains, and 3) what respondent characteristics are associated with awareness of racial, educational, and income disparities in health.

**Methods:**

We conducted the National Opinion Survey on Health and Health Disparities with 2,791 US adults. We asked respondents to answer questions about disparities in health between 1 of several pairs of population subgroups: African Americans versus whites, non–high school graduates versus high school graduates, high school graduates versus college graduates, the poor versus the middle class, or the middle class versus the rich. We used χ^2^ tests and logistic regression to compare correlates of respondents' awareness of disparities across the different pairs of population subgroups.

**Results:**

Most respondents were aware of health disparities between the poor and middle class (73%); fewer were aware of health disparities between African Americans and whites (46%). Although respondents recognized that education is associated with many positive life outcomes, they were less aware of the link between education and health. Respondents who were younger, less educated, lower-income, healthier, or politically conservative were less likely to be aware of health disparities.

**Conclusion:**

Public awareness of disparities in health differs depending on both the type of disparity and the characteristics of the individual respondent.

## Introduction

A key goal of *Healthy People 2020* is to eliminate health disparities (1) because overwhelming evidence indicates that disparities in health by race, socioeconomic status, and other demographic and social factors are large and persistent in the United States (2-5).

However, the general public may be unaware of these health disparities. A lack of public awareness may be a barrier to policy action aimed at addressing health disparities (6). Several recent initiatives aim to increase awareness of these health disparities. The PBS series *Unnatural Causes*, for example, is a documentary exploring racial/ethnic and socioeconomic inequalities in health, with a particular emphasis on the socioeconomic gradient in health, and the Robert Wood Johnson Foundation's Commission to Build a Healthier America addresses the nonmedical determinants of health and health disparities (2,7).

Despite these and other attempts to increase public awareness of health disparities, little is actually known about the US public's level of awareness. Although there is information about the public's awareness of disparities in health care in the United States (8,9), little information is available on the public's awareness of disparities in health outcomes. One exception is a study by Lillie-Blanton and colleagues, who conducted a survey in 1999 asking about respondents' awareness of differences between African Americans and whites in terms of infant death rates and life expectancy (9). They found that a majority of Americans (both white and African American) were unaware that African Americans are disadvantaged in these 2 health outcomes.

To better understand the public's awareness and opinions about the determinants of health and health disparities, we conducted the National Opinion Survey on Health and Health Disparities, building on an earlier survey conducted in Wisconsin (10). We present results from this nationwide survey and address 3 questions: 1) Is the US public aware of racial, educational, and income disparities in health? 2) Does awareness differ across these health disparity domains and across the socioeconomic gradient? 3) What respondent characteristics are associated with awareness of racial, educational, and income disparities in health?

## Methods

We contracted with the National Opinion Research Center to complete a random-digit–dialed telephone survey between November 2008 and February 2009. We selected a nationally representative sample and oversampled counties where at least 40% of the population is Hispanic or African American, at least 20% of the population lives below the federal poverty level, or at least 40% of householders have less than a high school education (to have a robust sample of low–socioeconomic status and racial/ethnic minority groups). We completed interviews with 2,791 respondents (28% from the oversample). We achieved a Council of American Survey Research Organizations response rate of 28%; the screener completion rate was 79%, and the interview rate was 46%. We created weights to account for differential probability of selection, unresolved telephone numbers, nonresponse, and multiple telephone and landline coverage, and to represent the national adult distribution by age, sex, and race. The Social Studies Institutional Review Board at the University of Wisconsin-Madison approved the project.

We based the survey instrument on an instrument from an earlier study in Wisconsin (10) and pilot tested before implementation. We constructed 7 panels to examine opinions about different types of known health disparities (2) and randomly assigned respondents to 1 of these panels. The surveys administered to all panels were identical except for questions about disparities. For these questions, the question structure was the same across all panels, except that we asked respondents to consider differences between 2 comparison groups based on 1) race (African Americans [Group A] vs whites [Group B]), 2) education (people who did not graduate from high school [Group A] vs people who graduated from high school [Group B], high school graduates [Group A] vs college graduates [Group B], and those without a college degree [Group A] vs those with a college degree [Group B]), 3) income (the poor [Group A] vs the middle class [Group B], the middle class [Group A] vs the rich [Group B], and those with low income [Group A] vs those with middle income [Group B]).

We asked respondents whether they think Group A is better off, just as well off, or worse off than Group B in terms of a number of life domains, including how healthy they are, getting medical treatment or health care when needed, having health insurance, getting a quality education, housing, opportunities for employment, household income, and overall quality of life. We then asked respondents who answered "worse off" to elaborate their answer as "somewhat worse off" or "much worse off." We defined awareness of a disparity as an initial answer of "worse off."

This survey design allowed us to identify whether awareness of health disparities differed simply on the basis of the groups being compared. By randomizing each respondent to only 1 disparity survey panel, we avoided potential contamination between answers that could occur if a respondent was asked sequentially about multiple types of health disparities rather than about just 1.

We classified respondent household income as less than 200% of the federal poverty level or at least 200%, race/ethnicity as Hispanic, non-Hispanic black, or non-Hispanic other; and education as college graduate, some college, or high school education or less. We asked respondents whether their overall health was excellent, very good, good, fair, or poor and to self-define political ideology as conservative, moderate, or liberal.

We conducted χ^2^ tests to examine whether awareness of health disparities differed across the survey panels and to examine bivariate relationships between respondent characteristics and awareness. We then used multivariate logistic regression to examine which respondent characteristics predicted awareness of each type of health disparity. We included variables in the logistic regression models if they were significantly associated (*P* < .05) with awareness in any of the survey panels. We conducted analyses using SPSS PASW release 18.0.0 (SPSS, Inc, Chicago, Illinois).

## Results

Respondents' characteristics were similar to those of the US population except that they were better educated (Table 1). There were no significant differences in the demographic characteristics of the randomized panels, indicating the randomization was successful and the panels can be compared directly.

### Awareness of disparities

We report results from 5 of the 7 survey panels because results for the panel about the poor versus the middle class were similar to results for the panel about low-income versus middle-class people, and results for the panel about high school graduates versus college graduates were similar to those for the panel about people without a college degree versus people with a college degree.

The percentage of respondents aware of health disparities was different across the 5 survey panels (African Americans vs whites, non–high school graduates vs high school graduates, high school graduates vs college graduates, the poor vs the middle class, and the middle class vs the rich) (Table 2). Almost half of respondents (46%) asked about African Americans versus whites were aware that African Americans are worse off than whites in terms of how healthy they are. Sixty percent of respondents recognized the existence of health disparities between people who did not graduate from high school and high school graduates, but only 35% were aware of health disparities between high school graduates and college graduates. Seventy-three percent of respondents recognized health disparities between the poor and the middle class, but only 44% were aware of health disparities between the middle class and the rich.

Respondents who were asked about education disparities were less likely to be aware of education disparities in health than they were to be aware of education disparities in other domains (Table 2). In contrast, those asked about disparities between the poor and middle class were as aware of income disparities in health (73%) as they were aware of income disparities in nearly all other domains.

### Respondent characteristics associated with awareness of health disparities

Awareness of health disparities between African Americans and whites ranged from a low of 32% (for conservative respondents and those with a high school education or less) to a high of 65% (among liberals) (Table 3). Higher percentages of respondents with education beyond high school, more income, or liberal ideology reported awareness of health disparities between African Americans and whites.

Awareness of health disparities between people who did not graduate from high school and those who graduated from high school ranged from 45% (among respondents with a high school education or less) to 71% (among college graduates). Both higher education and income were associated with awareness of health disparities for this education panel. In contrast, for respondents asked about disparities between high school graduates and college graduates, respondent education, income, age, and political ideology were associated with awareness of health disparities. Awareness of these disparities ranged from 19% (among respondents with a high school education or less) to 50% (among college graduates and liberals).

Respondents asked to focus on disparities between the poor and the middle class had the highest prevalence of awareness of health disparities; the range was from 47% (Hispanics) to 85% (liberals). The range of awareness of health disparities between the middle class and the rich was from 32% (Hispanics) to 64% (those in fair or poor health). Respondents with lower income, worse self-rated health, and liberal political ideology had greater awareness.

Many of the associations identified in the bivariate analyses remained significant in the multivariate analysis (Table 4). Overall, in multivariate models, respondent education level and political ideology were the strongest predictors of health disparity awareness. Respondents aged 65 years or older were twice as likely as younger adults to be aware of racial and educational health disparities. Similarly, college graduates were 1.5 to 3.9 times more likely than those with a high school education or less to be aware of racial and educational health disparities. Respondents with a college education were approximately 4 times as likely to be aware of health disparities between high school and college graduates. Liberals were approximately 3 times more likely than conservatives to be aware of health disparities by race, education (high school graduates vs college graduates), and income. Those with less income and worse health and liberals were more likely to be aware of health disparities between the middle class and the rich. Those in fair or poor health were more than twice as likely to be aware of health disparities between African Americans and whites as those in better health.

## Discussion

We found that awareness of racial health disparities was considerably lower than awareness of disparities between non–high school and high school graduates and the poor and middle class. These findings are consistent with the larger gaps in health seen between the different income and education levels than between racial/ethnic groups (2).

There was a notable lack of awareness of the socioeconomic gradient in health: 73% were aware that the poor have worse health than the middle class, but only 44% were aware that the middle class has worse health than the rich. Similarly, compared with the 60% who were aware of health disparities between people who did not graduate from high school and those who did, only 35% were aware of health disparities between high school graduates and college graduates. In other words, a majority of the public is aware that people at the bottom of the socioeconomic distribution have worse health than those above them, and a minority of the public recognizes that people at the top of the socioeconomic distribution have better health than those just below them. This finding reflects a general lack of awareness that a socioeconomic gradient in health exists across the entire socioeconomic distribution and is not disadvantageous only to people at the very bottom (11). Indeed, the varying results for the 2 education and 2 income panels emphasize the importance of choosing carefully which socioeconomic comparison groups to ask about when examining public opinion on socioeconomic disparities in health.

Although people are aware that education affects many life domains (eg, housing, income, access to health care), fewer are aware that education also affects health. In addition, less than half of the public recognizes that health disparities exist between African Americans and whites. These findings are similar to those of Lillie-Blanton and colleagues that 45% of whites and 42% of African Americans were not aware of black-white disparities in infant death rates and that 43% of whites and 46% of African Americans were not aware of black-white disparities in life expectancy (9).

Given evidence of the links between education and health (12,13) and of racial/ethnic disparities in health (5), our results suggest there may be a role for informational campaigns to raise awareness of educational and racial/ethnic disparities in health. However, research also demonstrates that communicating about the social determinants of health and health disparities is complicated because some methods of communication may heighten blame rather than concern for the group experiencing worse health (14,15). Moreover, increasing the public's awareness of health disparities does not guarantee that people will be concerned about or want to act on those disparities, although Rigby and colleagues found that awareness of health disparities was associated with support for government intervention to address these disparities (16).

Our results also show differences in awareness of health disparities by individual characteristics. Multivariate results demonstrate that respondents' political ideology is strongly associated with awareness of health disparities; liberals were approximately 3 times as likely as conservatives to be aware of racial and socioeconomic health disparities. Respondents with a college education were much more likely to be aware of health disparities by education level and health disparities between African Americans and whites. This finding is consistent with those of European studies, in which disadvantaged respondents appear less likely to recognize health inequalities (17,18).

In the multivariate analyses, age was a predictor of awareness of educational and race disparities in health. For example, respondents aged 65 years or older were more than twice as likely as 18- to 44-year-olds to be aware of health disparities between African Americans and whites. These individuals would have been young adults during the height of the civil rights movement, perhaps sensitizing them more to issues of racial disparities than younger Americans who did not witness this formative time in history.

Political analysis of policy preferences suggests that whether people are aware of "policy-specific facts" related to a particular issue influences their political views toward policies (19). Public awareness of health disparities is 1 such "policy-specific fact" that could influence public support for programs and policies to reduce health disparities. The high prevalence of public awareness of health disparities between the poor and the middle class suggests that if policy discussions arise regarding the reduction of health disparities specifically between the poor and middle class, much of the public will already recognize that these health disparities do, in fact, exist. In contrast, the low awareness of health disparities between African Americans and whites and by educational level may present a substantial challenge for efforts to address racial/ethnic and educational health disparities in the United States.

This study has several limitations. As with all telephone surveys, we did not reach all people randomly selected to participate. Although we oversampled disadvantaged neighborhoods, our sample was better educated than the general population. We used weighting to adjust for differential response. However, given our finding that better educated respondents are more aware of disparities, our overall levels of awareness may be overstated. Another potential limitation is that we refer to acknowledgment of health disparities as "awareness" of disparities, although some people's responses to these questions may reflect different cognitive processes. For example, those with conservative ideology, who on average were less likely to acknowledge health disparities, may be well aware that society believes these disparities to exist, yet do not themselves believe this to be true. Also, by assessing disparities by separate domains of race and socioeconomic status, we were implicitly assuming that people think about these domains discretely, which may not be the case.

In conclusion, we found that public awareness of disparities in health depends on both the type of disparity (awareness of income disparities was greater than that for racial or educational disparities) and the characteristics of the individual respondent. Understanding these differences in public awareness is necessary to shape policy actions to reduce disparities in health.

## Figures and Tables

**Table 1:** Respondent Characteristics, National Opinion Survey on Health and Health Disparities, 2008-2009

**Characteristic**	Respondents, % (n = 2,791)	US Adult Population,^a^ %
**Sex**		
Male	47	48
Female	53	52
**Age, y**		
18-44	48	49
45-64	36	34
≥65	16	17
**Race/ethnicity**		
Hispanic	11	14
Non-Hispanic black	11	11
Non-Hispanic other	78	75
**Education**		
High school or less	33	45
Some college	25	28
College graduate	43	27
**Region**		
Northeast	18	19
Midwest	27	22
South	35	37
West	20	23

a Source: 2000 US Census.

**Table 2:** Percentage of Respondents Reporting That Group A Is "Worse Off" Than Group B, by Different Domains of Potential Disparities, National Opinion Survey on Health and Health Disparities, 2008-2009

Domain	Groups Being Compared				

	African Americans (A) vs Whites (B), % (n = 373)	**Non**–**High School Graduates (A) vs High School Graduates (B), % (n = 378)**	High School Graduates (A) vs College Graduates (B), % (n = 388)	Poor (A) vs Middle Class (B), % (n = 391)	Middle Class (A) vs Rich (B), %(n = 378)
The groups differ in terms of their health.	46	60	35	73	44
One group is much worse off than the other.	13	26	10	36	19
One group is somewhat worse off than the other.	33	34	25	37	25
The groups differ in terms of getting medical treatment or health care when needed.	49	65	45	69	67
The groups differ in terms of having health insurance.	52	75	55	75	76
The groups differ in terms of getting a quality education.	47	NA	NA	64	66
The groups differ in terms of housing.	57	77	64	86	70
The groups differ in terms of employment opportunities.	44	90	74	71	67
The groups differ in terms of household income.	60	87	77	NA	NA
The groups differ in terms of quality of life.	46	72	52	77	47

Abbreviation: NA, not asked.

**Table 3:** Awareness That Group A Has Worse Health Than Group B, by Respondent Characteristics, National Opinion Survey on Health and Health Disparities, 2008-2009

Characteristic	Groups Being Compared									

	African Americans (A) vs Whites (B) (n = 373)		**Non**–**High School Graduates (A) vs High School Graduates (B)** (n = 378)		High School Graduates (A) vs College Graduates (B) (n = 388)		Poor (A) vs Middle Class (B) (n = 391)		Middle Class (A) vs Rich (B) (n = 378)	

	%	*P* Value	%	*P* Value	%	*P* Value	%	*P* Value	%	*P* Value
**Total**	46	NA	60	NA	35	NA	73	NA	44	NA
**Age, y**										
18-44	39	.14	53	.11	24	.01	75	.90	37	.10
45-64	48		63		42		73		49	
≥65	52		65		36		72		48	
**Sex**										
Male	50	.23	63	.30	35	.98	76	.27	45	.90
Female	43		58		35		71		44	
**Race/ethnicity**										
Hispanic	54	.19	65	.81	34	.39	47	.03	32	.14
Non-Hispanic black	57		63		44		80		56	
Non-Hispanic other	44		60		34		74		43	
**Education**										
High school or less	32	.001	45	<.001	19	<.001	65	.13	50	.23
Some college	48		59		30		73		43	
College graduate	57		71		50		77		40	
**Annual household income^a^**										
<200% poverty level	40	.006	49	<.001	26	<.001	63	.10	59	.001
≥200% poverty level	53		68		42		76		37	
Missing	32		46		16		76		46	
**Overall health**										
Excellent, very good, good	43	.05	61	.89	35	.39	74	.40	39	<.001
Fair or poor	56		56		36		69		64	
**Political ideology**										
Conservative	32	<.001	60	.36	28	<.001	68	.01	35	<.001
Moderate	47		58		36		69		38	
Liberal	65		67		50		85		62	

a Calculated on the basis of 2008 federal poverty levels and household size.

**Table 4:** Odds of Reporting That Group A Has Worse Health Than Group B, by Respondent Characteristics, National Opinion Survey on Health and Health Disparities, 2008-2009^a^

Characteristic	Groups Being Compared				

	African Americans (A) vs Whites (B), OR (95% CI) (n = 337)	**Non**–**High School Graduates (A) vs High School Graduates (B), OR** (95% CI) (n = 356)	High School Graduates (A) vs College Graduates (B), OR (95% CI) (n = 363)	Poor (A) vs Middle Class (B), OR (95% CI) (n = 370)	Middle Class (A) vs Rich (B), OR (95% CI) (n = 351)
**Age, y**					
18-44	1 [Reference]					
45-64	1.5 (0.9-2.7)	1.9 (1.1-3.3)	1.7 (0.9-3.2)	0.9 (0.5-1.6)	1.6 (1.0-2.7)
≥65	2.2 (1.1-4.2)	2.4 (1.3-4.5)	2.1 (1.1-4.2)	1.0 (0.5-2.0)	1.6 (0.8-3.0)
**Education**					
High school or less	1 [Reference]					
Some college	2.0 (1.0-4.1)	1.6 (0.9-2.9)	2.2 (1.0-3.9)	1.6 (0.8-3.1)	0.9 (0.5-1.7)
College graduate	2.6 (1.4-5.0)	2.6 (1.5-4.4)	3.9 (2.0-7.6)	1.5 (0.8-2.8)	1.0 (0.6-1.8)
**Annual household income^b^**					
≥200% poverty level	1 [Reference]					
<200% poverty level	0.8 (0.4-1.5)	0.7 (0.4-1.2)	0.6 (0.3-1.0)	0.6 (0.3-1.2)	1.9 (1.1-3.3)
Missing	0.5 (0.2-1.1)	0.4 (0.2-0.8)	0.2 (0.1-0.6)	1.1 (0.5-2.3)	1.1 (0.5-2.2)
**Overall health**					
Excellent, very good, good	1 [Reference]					
Fair or poor	2.1 (1.1-4.2)	0.8 (0.4-1.3)	1.4 (0.7-2.8)	1.0 (0.5-1.9)	2.4 (1.3-4.4)
**Political ideology**					
Conservative	1 [Reference]					
Moderate	1.5 (0.9-2.7)	0.7 (0.4-1.3)	1.6 (0.9-2.8)	1.1 (0.6-1.9)	1.0 (0.6-1.7)
Liberal	2.8 (1.5-5.1)	1.0 (0.6-1.8)	2.7 (1.4-5.1)	2.7 (1.3-5.3)	3.1 (1.8-5.5)

Abbreviations: OR, odds ratio; CI, confidence interval.

a Logistic regression models include all variables that were significant (*P* < .05) in the bivariate analyses for any survey panel.

b Calculated on the basis of 2008 federal poverty levels and household size.
